# Differences in clinical course between pituitary apoplexy and non-apoplexy patients with nonfunctioning pituitary neuroendocrine tumors

**DOI:** 10.1007/s10143-026-04172-6

**Published:** 2026-02-25

**Authors:** Akira Taguchi, Yasuyuki Kinoshita, Atsushi Tominaga, Fumiyuki Yamasaki, Vishwa Jeet Amatya, Yukio Takeshima, Nobutaka Horie

**Affiliations:** 1 Department of Neurosurgery, Graduate School of Biomedical and Health Sciences, Hiroshima University, 1-2-3, Kasumi, Minamiku, Hiroshima, 734-8551 Japan; 2 Department of Neurosurgery and Neuro-Endovascular Therapy, Hiroshima Prefectural Hospital, Hiroshima, Japan; 3 Department of Pathology, Graduate School of Biomedical and Health Sciences, Hiroshima University, Hiroshima, Japan

**Keywords:** Pituitary apoplexy, Nonfunctioning pituitary neuroendocrine tumor, Pituitary adenoma

## Abstract

Although the imaging diagnosis of pituitary apoplexy has been well reported, research on the clinical symptoms of this condition remains scarce. This study aimed to provide an accurate clinical evidence of patients with pituitary apoplexy. This retrospective study used data from 311 patients with nonfunctioning pituitary neuroendocrine tumors treated with initial transsphenoidal surgery. Pituitary apoplexy was defined as the presence of acute headache or visual impairment along with a tumor with pathological findings of necrotic changes and/or diffuse hemorrhage. To alleviate the effect of tumor volume and patient sex, propensity score matching was performed between the pituitary apoplexy (APO) and non-apoplexy (non-APO) groups. A total of 20 patients were matched to each of the two groups, and their data were statistically compared. Compared with the non-APO group, the APO group had higher preoperative body temperature (degrees Celsius) and lower levels of preoperative serum sodium (mEq/L) and prolactin (ng/mL). Receiver operating characteristic curve analysis revealed that the optimal prolactin level that supports the diagnosis of pituitary apoplexy was 7.5 ng/mL (AUC = 0.75250, 50% sensitivity, 100% specificity). The patients in the APO group were further divided into two subgroups, those who were evaluated within 3 days of onset and those who were evaluated after 3 days of onset, and the former subgroup was found to have lower serum PRL and sodium level as well as higher body temperature. Hyperthermia, hyponatremia, and hypoprolactinemia indicate the possibility of pituitary apoplexy.

Trial number: E-2020-2022, June 1, 2022, retrospectively registered.

## Introduction

Pituitary neuroendocrine tumors (Pit-NETs) are among the most common primary brain tumors encountered clinically [1]. They are categorized as functioning or nonfunctioning Pit-NETs, with the latter accounting for 14%–54% of all cases [2]. Nonfunctioning Pit-NETs cause visual impairment owing to the mass effect of the tumor and/or hormonal deficiency [3]. Pituitary apoplexy is a clinical syndrome resulting from abrupt hemorrhage and/or infarction of the pituitary gland, generally in a Pit-NET [4]. The clinical presentation is characterized by a rapid onset such as headache as the main symptom [5]. Pituitary apoplexy is characterized by a rapid increase in tumor volume, which requires consideration of early surgical resection. The UK guidelines recommend surgical treatment if severely reduced visual acuity, severe and persistent visual field defects, or deteriorating consciousness level is present [6]. Randeva et al. reported that patients who undergo transsphenoidal surgery (TSS) within 8 days of onset are more likely to recover their vision compared with patients [7]. In clinical practice, it is sometimes unclear whether symptoms have suddenly appeared in some patients. Pituitary apoplexy that has occurred before several days may show various signal intensities on magnetic resonance imaging (MRI) and may not exhibit a clear hyperdense lesion on computed tomography (CT) [4]. Subclinical hemorrhage on nonfunctioning Pit-NETs has a reported incidence of 22.3% and is more frequently observed in larger adenomas [8]. Therefore, surgeons are sometimes hesitant to decide whether to perform an early surgery and/or speculate whether the symptoms are caused by pituitary apoplexy.

This study aimed to investigate the clinical symptoms of patients with pituitary apoplexy and to provide new insights that may aid in the diagnosis of pituitary apoplexy.

## Materials and methods

This retrospective study was approved by the review board of Hiroshima University (E-2020-2022). The requirement for written informed consent was waived owing to the retrospective nature of the study.

### Patients

This study included 311 patients with nonfunctioning Pit-NETs who underwent initial TSS at our institution between 2009 and 2024. Their data, including age, sex, clinical symptoms, tumor volume, radiographical findings, and blood test results, including endocrinological and pathological findings, were obtained from their medical records and analyzed. In addition, their preoperative body temperature was recorded. The patients were divided into the pituitary apoplexy (APO) and non-apoplexy (non-APO) groups according to the presence or absence of acute clinical symptoms as well as necrotic changes and/or diffuse hemorrhage in the histopathological findings. The APO group consisted mostly of emergency patients; thus, the body temperature was measured on the day of the blood test. In one patient, the body temperature could not be measured at the time of the initial consultation; thus, it was measured on the day of surgery, as in the non-APO group.

### Endocrinological studies

In the non-APO group, the basal serum/plasma levels of thyroid-stimulating hormone (TSH), free thyroxine, prolactin (PRL), luteinizing hormone (LH), follicle-stimulating hormone, cortisol, adrenocorticotropic hormone (ACTH), growth hormone, and insulin-like growth factor 1 were measured approximately within 1 month preoperatively. In the APO group, the serum/plasma levels of pituitary hormone were measured at the time of or the morning after admission to our hospital. Patients who were transferred from other hospitals already received hydrocortisone. Pituitary provocation tests (PPTs) were conducted in almost all patients 3 months postoperatively. Two patients in the APO group and 29 in the non-APO group did not consent to undergo PPT and were thus lost to follow-up, or had a clear pituitary hormonal dysfunction.

### Radiological assessment

Almost all patients in the non-APO group underwent 3T-MRI and head CT within 1 month preoperatively for tumor volume and shape assessment. In the APO group, 11 patients underwent radiological assessment within 7 days of pituitary apoplexy onset; 7 patients, within 1 month of onset; and 2 patients, more than 1 month of onset. The tumor volume (cm^3^) was calculated as width × height × depth/2 [9]. Lateral tumor growth into the cavernous sinus was categorized according to the Knosp classification [10].

### Surgical procedures and timing of surgery for pituitary apoplexy

TSS was performed endoscopically through the nostril, as previously described [11]. In the APO group, the average day from pituitary apoplexy onset to surgery was 14, with a median day of 6 (interquartile range [IQR]: 3–15 days). Nineteen patients underwent surgery within 3 days from MRI evaluation, and one patient who had no headache underwent surgery at 16 days from MRI evaluation.

### Statistical analyses

The Shapiro–Wilk test was employed to assess data normality; continuous variables were expressed as median [IQR], whereas categorical variables were expressed as frequency (%). The Kruskal–Wallis test and one-way analysis of variance were employed to compare continuous variables, whereas Fisher’s exact test was used for categorical variables. A *P*-value < 0.05 (two-tailed) was deemed significant. Pituitary apoplexy is reportedly more common in men [5]. To exclude the influence of sex and tumor volume on clinical symptoms and pituitary hormone levels, propensity score matching was also performed. To estimate the propensity score, we fitted a logistic regression model for sex and tumor volume (cm^3^). One-to-one matching was completed using the nearest-neighbor match on the logit of the propensity score for pituitary apoplexy with the caliper width set to 0.20 times the standard deviation of the logit of the propensity score (random number seed: 111). A total of 20 propensity score-matched patients were classified as the matched non-APO group. Receiver operating characteristic (ROC) curves were created to determine the sensitivity and specificity of various cutoff values in the APO and matched non-APO groups for the serum PRL level. All statistical analyses were conducted using JMP^®^ version 16 (SAS Institute Inc., Cary, NC, USA).

## Results

### Before data matching

Table 1 presents the comparison data between the APO (*n* = 20) and non-APO (*n* = 291) groups before matching. The APO group had a higher proportion of men (*P* = 0.0003) and larger tumor volumes (*P* = 0.0169) than the non-APO group. Furthermore, the APO group had a higher incidence of headache (*P* < 0.0001). The non-APO group did not have patients with double vision (*P* = 0.0022). In addition, the APO group had lower serum levels of TSH (*P* = 0.0093), PRL (*P* = 0.0012), and LH (*P* = 0.0257) as well as higher preoperative body temperature (*P* = 0.0005) than the non-APO group. The APO group also had lower preoperative serum sodium levels (*P* < 0.0001).

**Table 1:** Comparison data between the APO (*n* = 20) and non-APO (*n* = 291) groups before matching

		Non-APO		APO		*P*-value
*n* = 311		291		20		
Age, y.o., median	(IQR)	61	(50–70)	61	(51–66)	0.5137
Sex	F:M	129:162		1:19		0.0003
Hypertension	(%)	89	(30.6%)	10	(50.0%)	0.0842
Diabetes mellitus	(%)	36	(12.4%)	3	(15.0%)	0.7259
Dyslipidemia	(%)	86	(29.6%)	4	(20.0%)	0.4517
Headache	(%)	30	(10.3%)	18	(90.0%)	<0.0001
Double vision	(%)	0	(0.0%)	6	(30.0%)	0.0022
Knosp grades 0–2	(%)	201	(69.1%)	16	(80.0%)	0.4505
Tumor volume, cm^3^, median	(IQR)	4.16	(2.18–8.46)	6.10	(4.52–14.64)	0.0169
Tumor with hyperdense lesion on CT	(%)	25	(9.4%)	16	(84.2%)	<0.0001
Thickening of the mucosa of the sphenoid sinus	(%)	1	(0.9%)	9	(47.4%)	<0.0001
Tumor with cyst	(%)	108	(37.1%)	14	(70.0%)	0.0075
Fluid–fluid level in the cyst	(%)	44	(40.7%)	7	(50.0%)	0.5714
Preoperative serum FT4, ng/dL, median	(IQR)	1.1	(0.9–1.2)	1.1	(0.6–1.2)	0.3061
Preoperative serum TSH, µIU/mL, median	(IQR)	1.98	(1.31–3.10)	1.36	(0.39–2.08)	0.0093
Preoperative serum PRL, ng/mL, median	(IQR)	15.4	(9.6–28.8)	7.5	(2.7–23.6)	0.0012
Preoperative serum LH, mIU/mL, median	(IQR)	4.3	(2.5–8.3)	3.1	(0.9–5.0)	0.0257
Preoperative serum FSH, mIU/mL, median	(IQR)	9.7	(5.2–21.7)	8.3	(3.7–12.5)	0.1821
Preoperative serum cortisol, µg/dL, median	(IQR)	10.2	(7.4–14.1)	7.1	(3.4–16.9)	0.2591
Preoperative serum ACTH, pg/mL, median	(IQR)	27.8	(17.8–39.4)	21.6	(13.1–39.2)	0.1970
Preoperative serum IGF-1, ng/mL, median	(IQR)	98	(67–131)	97	(53–144)	0.9506
SD score for IGF-1	(IQR)	-1.0	(-1.8–-0.3)	-1.0	(-2.5–-0.2)	0.7917
Preoperative diabetes insipidus (DI)	(%)	2	(0.7%)	1	(5.0%)	0.1813
Preoperative body temperature, ℃, median	(IQR)	36.5	(36.4–36.7)	36.9	(36.5–37.4)	0.0005
Preoperative serum sodium level, mEq/l, median	(IQR)	140	(139–141)	137	(133–140)	<0.0001
Postoperative cortisol replacement	(%)	16	(5.6%)	6	(30.0%)	0.0013
Postoperative thyroxine replacement	(%)	21	(7.2%)	4	(20.0%)	0.0651
Postoperative DI	(%)	5	(1.7%)	2	(10.0%)	0.0680

### Matched data

Table 2 presents the matched data. The differences in sex (*P* = 1.0000) and tumor volume (*P* = 0.7868, standard mean difference of 0.0017) between the groups were adjusted. Tumors with hyperdense lesions on CT (*P* < 0.0001) and T2 hypointense spots on MRI (*P* = 0.0033) were more common in the APO group. This group also had more cases with marked thickening of the mucosa of the sphenoid sinus. Compared with the matched non-APO group, the APO group had lower serum levels of PRL (*P* = 0.0063) and ACTH (*P* = 0.0145). Furthermore, the APO group had higher preoperative body temperature (*P* = 0.0002) and lower preoperative serum sodium levels (*P* = 0.0141) than the matched non-APO group.

**Table 2:** Comparison data between the APO (n = 20) and matched non-APO (n = 20) groups

		Matched non-APO		APO		*P*-value
*n* = 40		20		20		
Age, y.o., median	(IQR)	67	(56–73)	61	(51–66)	0.0546
Sex	F:M	1:19		1:19		1.0000
Tumor volume, cm^3^, median	(IQR)	6.05	(4.54–14.23)	6.10	(4.52–14.64)	0.7868
Knosp grades 0–2	(%)	16	(80.0%)	16	(80.0%)	1.0000
Headache	(%)	1	(5.0%)	18	(90.0%)	<0.0001
Tumor with hyperdense lesion on CT	(%)	1	(5.0%)	16	(84.2%)	<0.0001
T1 hyperintense spot on MRI	(%)	6	(30.0%)	8	(42.1%)	0.5145
T1 hypointense spot on MRI	(%)	10	(50.0%)	4	(21.1%)	0.0958
T2 hyperintense spot on MRI	(%)	15	(75.0%)	18	(94.7%)	0.1818
T2 hypointense spot on MRI	(%)	9	(45.0%)	17	(89.5%)	0.0057
Thickening of the mucosa of the sphenoid sinus	(%)	1	(5.0%)	9	(47.4%)	0.0033
Preoperative serum TSH, µIU/mL, median	(IQR)	2.14	(1.05–3.21)	1.36	(0.39–2.09)	0.0884
Preoperative serum PRL, ng/mL, median	(IQR)	14.7	(10.2–33.5)	7.5	(2.7–23.6)	0.0063
Preoperative serum LH, mIU/mL, median	(IQR)	2.9	(1.7–5.1)	3.1	(0.9–5.0)	0.4244
Preoperative serum FSH, mIU/mL, median	(IQR)	7.8	(4.2–12.5)	8.3	(3.7–12.5)	0.8924
Preoperative serum cortisol, µg/dL, median	(IQR)	11.0	(5.4–12.7)	7.1	(3.4–16.9)	0.5978
Preoperative serum ACTH, pg/mL, median	(IQR)	33.7	(26.9–57.7)	21.6	(13.1–39.2)	0.0145
Preoperative serum IGF-1, ng/mL, median	(IQR)	75	(55–131)	97	(53–144)	0.3254
SD score for IGF-1	(IQR)	−1.5	(−2.5–-0.7)	−1.0	(−2.5–-0.2)	0.4562
Preoperative diabetes insipidus (DI)	(%)	1	(5.0%)	1	(5.0%)	1.0000
Preoperative body temperature, ℃, median	(IQR)	36.4	(36.3–36.6)	36.9	(36.5–37.4)	0.0002
Preoperative serum sodium level, mEq/L, median	(IQR)	140	(139–140)	137	(133–140)	0.0141
Postoperative cortisol replacement	(%)	1	(5.0%)	6	(30.0%)	0.0915
Postoperative DI	(%)	1	(5.0%)	2	(10.0%)	1.0000

Table 3 presents a comparison of the postoperative PPT results between the APO and matched non-APO groups. The APO group had lower basal serum levels of PRL (*P* = 0.0101) and peak PRL (*P* = 0.0083) than the matched non-APO group. However, no significant differences were observed in other pituitary hormonal secretions between the groups. Six patients (30.0%) in the APO group required postoperative cortisol replacement therapy.

**Table 3:** Comparison of the postoperative pituitary provocation test between the APO and matched non-APO groups

		Matched non-APO		APO		*P*-value
n = 40		20		20		
Serum TSH level, µIU/mL, median	(IQR)	2.44	(1.19–4.07)	2.04	(1.30–3.44)	0.5837
Peak TSH level, µIU/mL, median	(IQR)	13.50	(8.65–17.81)	11.09	(4.64–19.21)	0.7166
Serum PRL level, ng/mL, median	(IQR)	9.5	(6.0–15.0)	5.9	(3.9–8.9)	0.0101
Peak PRL level, ng/mL, median	(IQR)	30.9	(20.9–42.4)	14.3	(6.8–28.9)	0.0083
Serum LH level, mIU/mL, median	(IQR)	3.3	(1.4–6.1)	4.1	(2.0–6.3)	0.4822
Peak LH level, mIU/mL, median	(IQR)	12.3	(6.0–26.4)	13.3	(4.8–22.7)	0.8819
Serum FSH level, mIU/mL, median	(IQR)	5.4	(2.6–8.7)	7.4	(2.8–10.5)	0.4479
Peak FSH level, mIU/mL, median	(IQR)	7.3	(5.4–12.8)	11.6	(3.8–15.7)	0.8430
Peak GH level, ng/mL, median	(IQR)	1.51	(0.46–2.30)	1.15	(0.27–3.74)	0.8301
Serum cortisol, µg/dL, median	(IQR)	8.0	(6.7–10.0)	8.2	(5.7–10.1)	0.8551
Peak cortisol, µg/dL, median	(IQR)	13.3	(9.2–15.1)	16.2	(8.3–19.3)	0.3873
Serum ACTH, pg/mL, median	(IQR)	34.9	(22.6–51.7)	21.0	(12.9–36.8)	0.0542

### ROC for serum PRL levels

**Fig. 1:**
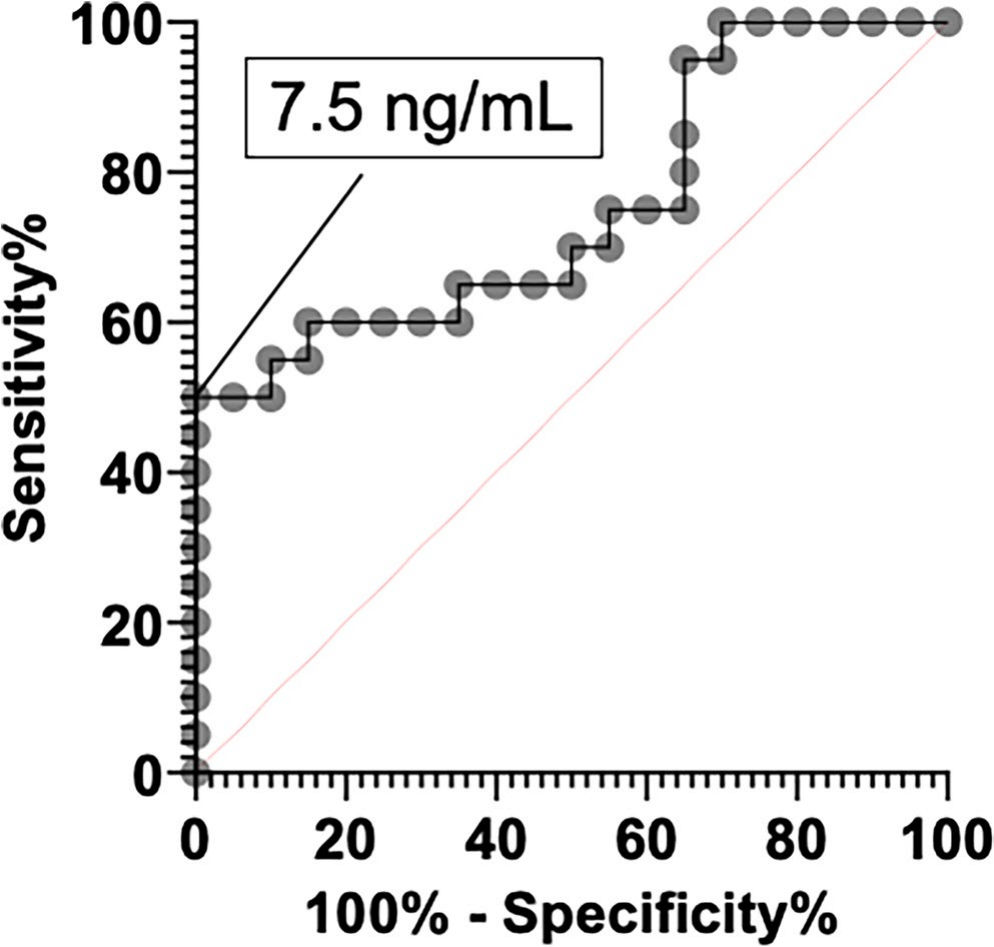
Sensitivity and specificity of serum PRL for the diagnosis of pituitary apoplexy. A total of 40 cases were evaluated, with tumor volumes ranging from 1.41 to 20.51 cm^3^.The optimal cutoff value of the serum PRL level was 7.5 ng/mL (i.e., 50.0% sensitivity, 100.0% specificity, AUC = 0.75250, *P* = 0.0063)

Figure 1 presents the sensitivity and specificity of serum PRL for the diagnosis of pituitary apoplexy. The optimal cutoff value of the serum PRL level was 7.5 ng/mL (i.e., 50.0% sensitivity, 100.0% specificity, AUC = 0.75250, *P* = 0.0063).

### Differences between PRL < 7.5 and ≥ 7.5 ng/mL in the APO group

The patients in the APO group were further divided into two subgroups based on whether the serum PRL level was more than 7.5 ng/mL (Table 4). The low PRL subgroup had higher preoperative body temperature (*P* = 0.0064) and lower serum sodium levels (*P* = 0.0010).

**Table 4:** Classification based on the preoperative serum PRL level of 7.5 ng/ml

Table		PRL < 7.5 ng/mL		PRL > 7.5 ng/mL		*P*-value
*n* = 20		10		10		
Age, y.o., median	(IQR)	60	(45–65)	62	(53–71)	0.3250
Sex	F:M	0:10		1:9		1.0000
Tumor volume, cm^3^, median	(IQR)	5.64	(4.59–10.19)	6.91	(3.80–16.71)	0.7055
Preoperative body temperature, ℃, median	(IQR)	37.3	(37.0–38.0)	36.6	(36.5–36.8)	0.0010
Preoperative serum sodium level, mEq/L, median	(IQR)	135	(133–137)	140	(138–141)	0.0070
Postoperative serum PRL level, ng/mL, median	(IQR)	4.1	(2.6–5.7)	8.9	(6.4–13.1)	0.0010
Postoperative serum IGF-1, ng/mL, median	(IQR)	78	(47–129)	82	(36–93)	0.8046
Postoperative serum cortisol, µg/dL, median	(IQR)	7.0	(3.0–10.3)	9.0	(7.8–10.4)	0.1736
Postoperative peak cortisol, µg/dL, median	(IQR)	15.2	(5.8–19.0)	16.2	(11.3–21.2)	0.4350
Postoperative serum ACTH, pg/mL, median	(IQR)	19.4	(10.3–30.9)	22.8	(11.3–42.7)	0.7055
Postoperative cortisol replacement	(%)	3	(30.0%)	3	(30.0%)	1.0000
Postoperative thyroxine replacement	(%)	2	(20.0%)	2	(20.0%)	1.0000

### Serum PRL level, sodium level, and body temperature evaluated by the days since pituitary apoplexy

**Fig. 2:**
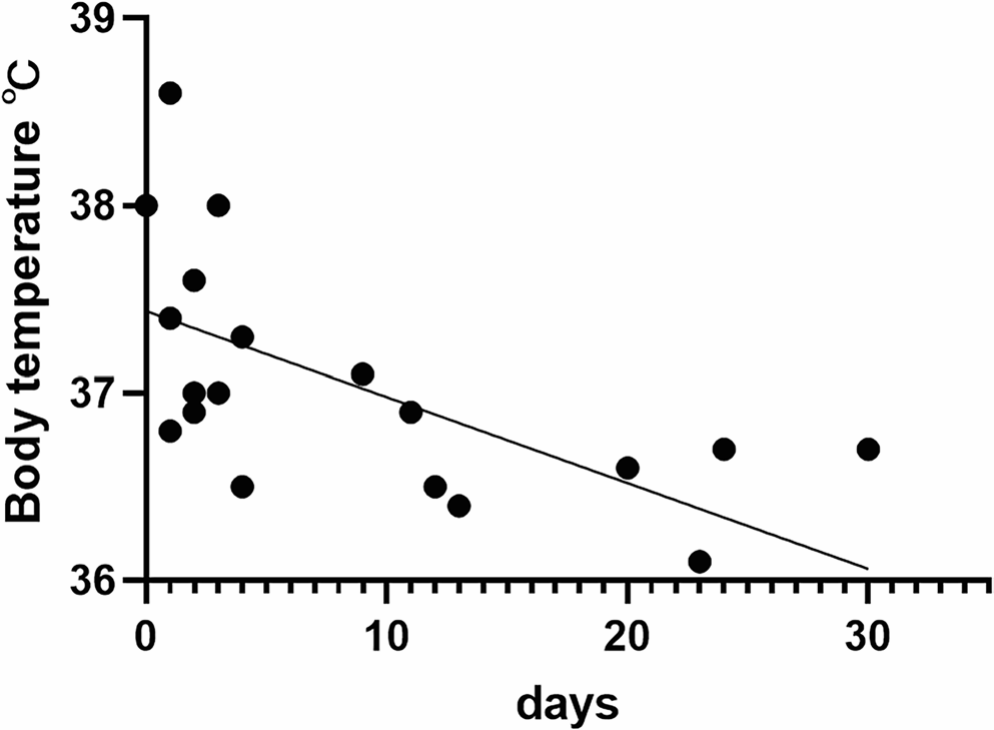
Correlation between body temperature on the day of pituitary apoplexy diagnosis and the number of days since onset. A weak negative correlation (*P* = 0.0009, R^2^ = 0.466528) with the number of days since onset was observed

In the present study, a variation in the time from pituitary apoplexy onset to patient evaluation at the hospital were observed. Therefore, the patients in the APO group were divided into two groups: those who were evaluated within 3 days of onset and those who were evaluated after 3 days of onset. The former subgroup had lower serum levels of PRL (*P* = 0.0012) and sodium (*P* = 0.0350) (Table 5). Body temperature exhibited a weak negative correlation (*P* = 0.0014, R^2^ = 0.442205) with the number of days since onset (Fig. 2).

**Table 5:** Classification based on blood test within 3 days of onset

Table		Onset day > 3		Onset day ≤ 3		*P*-value
*n* = 20		10		10		
Age, y.o., median	(IQR)	62	(53–71)	59	(43–64)	0.1483
Sex	F:M	1:9		0:10		1.0000
Tumor volume, cm^3^, median	(IQR)	6.10	(4.33–13.09)	5.65	(4.82–13.09)	0.9697
Preoperative serum PRL level, ng/mL, median	(IQR)	17	(8.1–29.2)	2.7	(1.5–5.5)	0.0012
Preoperative serum sodium level, mEq/L, median	(IQR)	139	(134–141)	136	(133–137)	0.0350

## Discussion

In this study, we compared the background characteristics between the APO and non-APO groups, with the former group having higher preoperative body temperature and lower serum levels of sodium and PRL. The three aforementioned parameters may be more clearly indicated early in the onset of pituitary apoplexy.

### Body temperature and pituitary apoplexy

Patients with pituitary apoplexy experience fever due to the following possible reasons: First is the presence of chemical meningitis caused by leakage of necrotic or hemorrhagic components from the tumor with apoplexy into the subarachnoid space [12–14]. Second is the possibility of adrenal insufficiency [15]. For example, in patients with infectious diseases, activated macrophages produce tumor necrosis factor-α (TNF-α), which in turn produces inflammatory mediators, such as IL-1 and IL-6. These mediators act on the hypothalamus, increasing prostaglandin levels and causing fever. Contrarily, inflammatory cytokines physiologically stimulate the hypothalamus–pituitary–adrenal (HPA) axis, leading to increased cortisol concentrations [16]. Hypercortisolemia suppresses TNF-α and IL-6 production [17]. Thus, in patients with pituitary apoplexy, tumor necrosis may induce local inflammatory cell infiltration and macrophage phagocytosis, inducing inflammatory cytokines, whereas fever may occur due to decreased ability to suppress inflammatory responses owing to insufficient cortisol secretion.

### Hyponatremia due to adrenal insufficiency

Corticotropic deficiency is one of the most commonly observed deficits in patients with pituitary apoplexy [4]. ACTH deficiency ultimately leads to hyponatremia through AVP-dependent and direct renal mechanisms [18]. In this study, hyponatremia was considered to have been caused by corticotropic deficiency. Patients who visited the emergency room or were urgently referred from other hospitals have already received hydrocortisone upon arrival at our hospital or at the time of blood sampling the following morning. Thus, the plasma ACTH and serum cortisol levels may not accurately reflect the endocrinological pathology.

### Clinical significance of hypoprolactinemia due to pituitary tumor

Nonfunctioning Pit-NETs with hyperprolactinemia due to the stalk effect are well known. In previous studies, 20%–27% of patients with pituitary apoplexy had PRL levels below 5 ng/mL [19, 20]. Thus, low serum PRL levels appear to be relatively in patients with pituitary apoplexy. Zayour et al. reported that the mean PRL value was lower in the large Pit-NET group with pituitary apoplexy than that without [21]. They speculated that the sudden and rapid increase in intrasellar pressure caused by pituitary apoplexy leads to ischemic necrosis of the anterior pituitary cells, resulting in reduced viability [21].

In the present study, the APO group had low serum PRL levels, whereas the non-APO group had normal or high levels. Therefore, the low PRL levels of patients with Pit-NETs despite relatively large tumors likely to have a stalk effect suggest the presence of pituitary apoplexy. Moreover, in the APO group, patients with PRL level of 7.5 ng/mL or less had lower serum sodium levels and higher preoperative body temperatures than those with PRL level of 7.5 ng/mL or more. As aforementioned, these findings indicate adrenal insufficiency and support the potential utility of low serum PRL levels as a predictive marker of anterior pituitary cell damage.

### Differences in the number of days from onset to diagnosis

In the present study, patients evaluated early after pituitary apoplexy onset tended to have lower serum prolactin and sodium levels and higher temperatures. Partial or complete recovery of pituitary function was observed in up to 50% of patients with pituitary apoplexy, and more recent retrospective studies reported no statistically significant differences in the endocrine outcome between surgically and conservatively managed patients [6]. These findings suggest that pituitary hormonal function may have improved over time in patients who presented with normal serum PRL and sodium levels and normal body temperature. In fact, some patients who visited after the onset of symptoms other than headache and visual field disturbance showed improvement. Because headache, low-grade fever, and fatigue are also common symptoms of viral infections, it is often difficult for both patients and clinicians to suspect early-stage pituitary dysfunction.

No correlation was observed between preoperative serum PRL levels and early postoperative recovery of the HPA axis. In pituitary apoplexy, inflammation associated with tumor ischemia is assumed to affect the pituitary cells and induce rapid compression of the pituitary gland due to sudden tumor enlargement. Subsequently, the intrasellar pressure gradually decreased due to the shrinkage caused by tumor necrosis. This process may contribute to the restoration of the HPA axis and influence the time course of serum PRL levels from the onset. However, these dynamic changes could not be clearly delineated in the present study.

### Limitations

This study has several limitations that should be acknowledged. First, a variation was observed in the time from pituitary apoplexy onset to patient evaluation at the hospital. Second, although the present study suggests that one of the underlying mechanisms contributing to the major clinical features of pituitary apoplexy is adrenal insufficiency, we were unable to accurately evaluate the plasma ACTH and serum cortisol levels. Furthermore, pituitary apoplexy is a rare disease, and the study included a small sample size. Therefore, further prospective studies are warranted to validate these findings. Despite the aforementioned issues, to our knowledge, this study is the first to demonstrate an association between pituitary apoplexy and body temperature. This study provides new insights into the clinical characteristics of pituitary apoplexy and may contribute to improved clinical recognition.

## Conclusion

In nonfunctioning Pit-NETs with acute onset symptoms, hyperthermia, hyponatremia, and hypoprolactinemia may indicate the possibility of pituitary apoplexy.

## Data Availability

The aggregated data generated and analyzed during the current study are not publicly available due to ethical restrictions and patient confidentiality but are available from the corresponding author on reasonable request.
